# Fate of PAHs in the vicinity of aluminum smelter

**DOI:** 10.1007/s11356-018-2648-0

**Published:** 2018-07-03

**Authors:** Jacek Borgulat, Tomasz Staszewski

**Affiliations:** Institute for Ecology of Industrial Areas, Kossutha 6, 40-844 Katowice, Poland

**Keywords:** PAHs, Aluminum smelter, Grass, Spruce, Soil

## Abstract

Investigation has been carried out in the vicinity of an aluminum smelter located in the industrialized town of Konin. Concentrations of 14 polycyclic aromatic hydrocarbons (PAHs) were determined in grass, spruce needles, and soil collected in the period of the smelter operation and several years after its closing. Significant changes in the quantity of PAHs and their profiles observed in the two measuring periods, stressing the importance of aluminum production with regard to PAH emission. It was confirmed by very high values of the carcinogenic potential (CP) found for PAHs accumulated in grass and soil when compared to the values found in urban and remote sites. PAH ratio rates used as a tool for identifying emission sources showed a pyrogenic origin of PAHs in both periods; the ratios in the period of the smelter running activity were similar to those found in other studies carried out near aluminum smelters. Grass turned out to be a good biomonitor of PAHs similarly to commonly used leaves of various tree species. The use of four age classes of spruce needles, some of which were subjected to emission from the smelter, showed that such approach could serve as an analysis tool for describing retrospective pollution.

## Introduction

Polycyclic aromatic hydrocarbons (PAHs) found in the environment can be of anthropogenic or natural origin (Stogiannidis and Laane 2015). Anthropogenic PAHs can be pyrogenic, if they originate from different pyrolysis substrates, such as fossil fuels and biomass, and petrogenic, if the come from petroleum-related sources (Boehm et al. 2007). It was estimated that the total anthropogenic atmospheric emission of 16 PAHs over the world in 2004 was 520 (Gg·year^−1^) with biofuel (56.7%), wildfire (17.0%), and consumer product usage (6.9%) as the major sources, which far exceeded natural sources (Zhang and Tao 2009). PAH emissions from Europe accounted for 9.5% of the total PAH emissions (Zhang and Tao 2009). According to Poland’s Informative Report 2017 (Dębski et al. 2017), in this country, the main source of PAH emission (87%) are “non-industrial combustion plants” (mostly residential heating plants), the second being “production processes” with coke production as the dominant subsector.

Due to their volatility and association with fine and ultra-fine particulates, PAHs may be transported far away from their original source and reach various environmental compartments. In most cases, PAHs are deposited on plant surface, on inorganic fraction of soils or in sediments (Stogiannidis and Laane 2015).

Direct collection and analyses of atmospheric samples can provide the most reliable data on PAH contamination of the atmosphere. This approach, however, has also some limitations; one of them lies in the fact that, according to this approach, data can be provided only for a limited number of sampling stations requiring power supply. Moreover, the data refer to a specific sampling period, and it is impossible to get information concerning the retrospective pollution (Krivan 1989).

The use of plants as passive samplers of PAHs in the atmosphere has been suggested by various authors to assess the degree of long-term pollution trends at any place where those bioindicators are available (Müller et al. 2001; Niu et al. 2003; De Nicola et al. 2005; Srogi 2007). Nevertheless, a list of factors may heavily affect the accumulation of chemical compounds in the bioindicator. PAHs transported in the air and deposited on plant and soil surface are subjected to various meteorological factors, like temperature (Franzaring 1997; Fang et al. 2006, Lin et al. 2011, Slezakova et al. 2013,), heavy rainfall (Lehndorff and Schwark 2004; De Nicola et al. 2008; Sun et al. 2010), high wind speed (Horstmann and McLachlan 1998; Haugen et al. 1999; Lee and Jones 1999; Chaloulakou et al. 2003) and sunlight; besides, they are prone to react with oxidizing gases, e.g., ozone (Finlayson-Pitts and Pitts 2000; Park et al. 2002; Tsapakis and Stephanou 2005; Tham et al. 2008; Slezakova et al. 2013). All these factors not only tend to lower the PAH loads, but also to modify the percent profile of PAHs released by direct sources.

Despite these limitations, some efforts have been undertaken to find proper indices of the PAH profile (PAH ratios and some of their combinations) that would allow source characterization of contaminated areas (Stogiannidis and Laane 2015).

Among the anthropogenic sources of PAHs, aluminum production is one of the principal ones. The use of the Söderberg process leads to “combustion” of coke anodes, with coal tar pitch acting as a binder. The PAHs are therefore volatilized at the process temperature, which is close to 1000 °C. The purpose of this research was to analyze the profile of emission from the Konin aluminum smelter on the basis of the monitoring of PAH content in grass, spruce needles, and soil in the vicinity of the smelter which has been carried out during its operation and after its shutdown.

## Material and methods

### Study area

Until the 1950s of the twentieth century, Konin was a small town with 12 thousand inhabitants and surrounded by agricultural areas. In this place, where previously, no industrial activity was performed, a complex of power stations combusting brown coal was built at the end of the 1950s, followed by the aluminum smelter which was put into operation in 1965, reaching the annual aluminum production of 43,000 tons. The town has developed rapidly and nowadays, it is a mean-size city hosting about 80,000 residents and intensive traffic mainly due to the arterial road leading to the seaside.

The operation of the aluminum smelter using the Söderberg process in the aluminum production with very poor emission control adversely impacted the surrounding agricultural and forest areas. It resulted in cutting off a big area from agricultural activity and the displacement of local people from the areas threatened by industrial emissions. Implementation of the principles of the state environmental policy established in 1991 extorted modernization of the production technology. After 1993, modernization the electrolysis process started. “Wet” anode mass was replaced with “dry” anode mass, in which the effect was lower PAH emission to environment. However, in February 2009—due to economic reasons—the smelter was shut down.

**Fig. 1:**
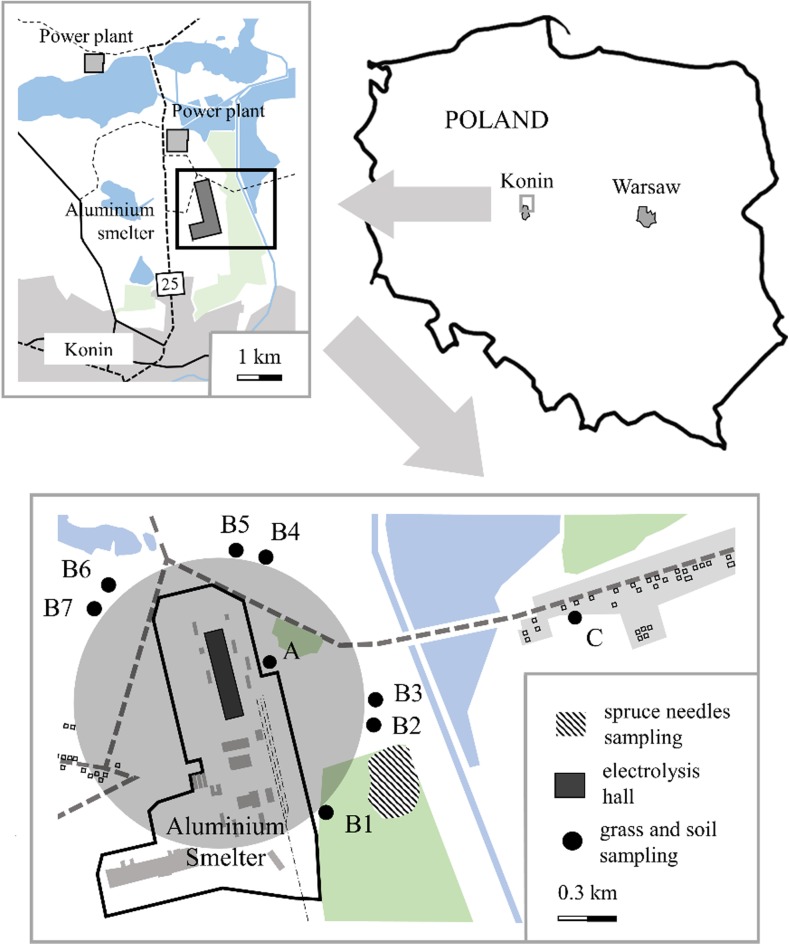
Location of sites

The investigated area is a wasteland located near to the complex of power stations and about 4 km from the town center. Nearby, there are dozens detached houses fired with brown coal or wood (Fig. 1).

### Sampling

The following sampling sites were chosen for our study (Fig. 1):

(A) 0.25 km from the emitter
(B) 1–7—seven sites within a radius of about 0.8 km of the emitter hereinafter referred to as site B
(C) site located 2 km from the emitter

Vegetation and soil material was collected in October 2007 and 2010 years. From each site, one composite sample containing grass was taken in the radius of 30 m. In site B, at three places, in 40-year-old spruce stands, the needles were sampled in standardized way from the seventh shoot whorls of six trees (Fig. 1). Needles were divided into four age classes: 2- and 3-year-old (both representing the smelter operation period), 1-year-old, and current year needles (representing the period after its shutdown). The composite samples of needles belonging to each age class were analyzed. The results are given as a mean of values obtained for the three places.

Vegetation samples were wrapped in aluminum foil and transported to the laboratory in a refrigerated box (4 °C). Then, they were stored at − 20 °C until analysis. Extraction procedure was carried out on defrosted matter. Ten grams of plant material from each sample was dried at 30 °C and ground for analyses.

Soil samples were collected from each site with the use of a hand-held twisting probe (Egner’s stick). Soil taken at the depth of 0–30 cm was used as material for analyses. Each time, 15 individual samples were taken thereby providing a mean mixed sample of about 1 kg. Dried soil samples were sieved (1-mm sieve) in the laboratory and used for further chemical characterizations.

### Chemical analyses

The extraction of vegetation and soil samples was performed with the use of dichloromethane using an ASE automatic extractor in accordance with the program (*T* = 100 °C, *p* = 10 Mpa). Ten milliliters of solvent was used for the extraction. For each sample, two cycles of extraction were performed. Further purification was carried out using the column chromatography (Florisil). The PAH content analysis in plant and soil material was performed by liquid chromatograph (Hewlett Packard HPLC 1050) with fluorescence detector. Analyses were conducted using Baker’s chromatographic column—BAKERBOND PAH-16 Plus (250 × 3 mm, 5 μm). Column temperature was isothermal at 27 °C. The following gradient elution (methanol/water) was used: 0–6 min, 75% methanol; 6–30 min, 100% methanol (flow 0.5 ml/min). For the PAH determination, the following detection parameters were used: 218/332 nm before phenanthrene, after 7.25 min 260/420 nm; before benzo[b]fluoranthene, after 12.5 min 290/420 nm; after 22 min, indeno[123-cd]pyrene 248/500 nm. Identification of compounds was carried out by the comparison of their retention times with the values obtained from a mixture of the Supelco PAH Mixture 610–M (Wild et al. 1992). Blind samples and plant samples fortified with a known amount of PAHs were added to the sample series to determine the PAH recovery rate.

All chemicals used were of analytical grade. The following polycyclic aromatic hydrocarbons (PAHs) listed by the United States Environmental Protection Agency (USEPA) and the European Community as priority pollutants were examined: naphthalene (Naph), fluorene (Fluo), phenanthrene (Phen), anthracene (Ant), fluoranthene (Flt), pyrene (Pyr), benzo[a]anthracene (BaA), chrysene (Chry), benzo[b]fluoranthene (BbF), benzo[k]fluoranthene (BkF), benzo[a]pyrene (BaP), dibenzo[ah]anthracene (DahA), benzo[ghi]perylene (BghiP), indeno[123-cd]pyrene (IcdP). The entire analytical procedure underwent quality control checks. Analyses of blanks were performed for every eight samples. PAH concentration in all blank values was below the detection limit. Recovery results varied between 81 and 96%.

The analyses were carried out in a certified laboratory of the Institute for Ecology of Industrial Areas, Katowice (Certificate No AB 325).

### Carcinogenic potential

In the studied samples, the carcinogenic potential (CP) index of the PAH deposit, proposed by us earlier (Mętrak et al. 2016) was calculated according to the formula (1) with the use of estimations of carcinogenic properties provided by the EPA Tasmania (2012) (Table 1).

1 $$ \mathrm{CP}=\frac{\sum \left({\mathrm{AMT}}_{\mathrm{PAH}}\times {\%}_{\mathrm{carc}\ \mathrm{vs}\ \mathrm{BaP}}\right)}{100} $$

**Table 1:** Characteristics of carcinogenic PAHs (EPA Tasmania 2012)

Compound (abbreviation, number of rings)	Carcinogenic hazard vs BaP [%]
Benzo[a]antracene (BaA, 4)	10
Chrysene (Chr, 4)	1
Dibenzo[ah]anthracene (DahA, 5)	109.09
Benzo[b]fluoranthene (BpF, 5)	10
Benzo[k]fluoranthene (BkF, 5)	10
Benzo[a]pyrene (BaP, 5)	100
Indeno[123-cd]pyrene (IcdP, 6)	10

AMT_PAH_: amount of carcinogenic PAH
%_carc vs BaP_: percent of carcinogenic properties of PAH vs BaP

### Statistical analysis

In order to determine differences between soil and plant samples collected from site B (B1–B7) in two measuring periods, we used the *t* test (*n* = 7, *p* < 0.05).

Cluster analysis (distance measure—Euclidean distance, agglomeration method—Ward’s method) was used to determine the similarity in the content of the selected PAHs in plant material. Due to their volatility, the 2- and 3-ring PAHs were rejected in the analysis.

In order to perform the calculations and construct the dendrogram, we used a StatSoft STATISTICA version 13.0 computer software (StatSoft Inc., Tulsa, USA).

## Results

### PAHs in grass

The contents of the analyzed PAHs in grass samples were presented in Table 2. In the period of the smelter operation, a concentration of 14 PAHs in grass collected near the emitter (site A) amounted to 9035 μg kg^−1^. The concentration decreased along with the distance from the smelter and was found to be 1591 and 1277 μg kg^−1^ in site B and C, respectively. In all sites, 4-ring PAHs prevailed in the grass making above 50% of the PAH sum. The share of 5- and 6-ring PAHs was similar in site A and C (35%) and in site B, it amounted to 25%. The CP index calculated on the basis of Eq. (1) for site A (909) was nine times higher than in other sites.

**Table 2:** Contents of PAHs in grass (μg kg^−1^)

Year	Site A		Site B				Site C
			Mean^c^	SD	Min.	Max.	
2007^a^	Naph	533	120	54	60	208	66
	Fluo	95	22	19	7	64	8
	Phen	463	163	69	102	301	105
	Ant	123	31**	18	18	70	18
	Flt	1547	383***	155	214	648	228
	Pyr	1135	226**	114	130	423	155
	BaA	634	88***	56	36	135	78
	Chry	1276	160**	136	49	276	192
	BbF	1174	153**	89	52	282	200
	BkF	402	53***	25	21	84	50
	BaP	459	46*	25	20	92	40
	DahA	139	16*	9	6	33	20
	BghiP	405	51**	33	19	90	67
	IcdP	650	79**	43	34	143	50
	∑_2-ring PAHs_	533 (5%)	120 (8%)	54	60	208	66 (6%)
	∑_3-ring PAHs_	681 (10%)	216 (14%)	104	132	435	131 (7%)
	∑_4-ring PAHs_	4592 (51%)	858*** (53%)	439	429	1482	653 (51%)
	∑_5-ring PAHs_	2174 (24%)	268** (16%)	139	103	448	310 (24%)
	∑_6-ring PAHs_	1055 (10%)	131** (9%)	74	53	233	117 (12%)
	∑ _PAHs_	9035	1591*				1277
	*CP*	909	102***	55	45	183	102
2010^b^	Naph	98	119	22	90	155	103
	Fluo	27	33	10	17	50	(0.9)
	Phen	67	111	23	85	141	73
	Ant	2	6**	3	3	11	8
	Flt	27	67***	18	47	89	57
	Pyr	23	43**	8	35	55	44
	BaA	1	11***	8	(0.9)	19	15
	Chry	13	24**	6	17	31	26
	BbF	3	14**	8	(0.9)	25	17
	BkF	2	3***	6	(0.9)	13	4
	BaP	15	24*	6	18	35	28
	DahA	3	5*	3	1	10	6
	BghiP	11	15**	8	(0.9)	25	19
	IcdP	8	18**	6	10	28	22
	∑_2-ring PAHs_	98 (33%)	119 (25%)	22	90	155	103 (24%)
	∑_3-ring PAHs_	96 (32%)	150 (30%)	23	125	187	81 (19%)
	∑_4-ring PAHs_	64 (21%)	145*** (29%)	36	102	186	142 (34%)
	∑_5-ring PAHs_	23 (8%)	46** (9%)	22	20	82	55 (13%)
	∑_6-ring PAHs_	19 (6%)	33** (7%)	13	10	53	41 (10%)
	∑ _PAHs_	300	493*				422
	*CP*	21	34***	12	22	54	40

^a^Samples affected by emission from aluminum smelter

^b^Samples collected after emission period

^c^Statistical significance between different years (*n* = 7, *t* test *p < 0.05, **p < 0.01, ***p < 0.001)

(%) Percentage values (*m*/*m*)

A completely different pattern of PAHs in the grass was observed 3 years after the smelter shutdown. Much lower PAH concentrations (except Naph, Fluo, and Phen) were found in grass growing at all investigated sites. Total PAHs concentrations were significantly less changing with sites than in the previous period, the maximum was observed in site B (493 μg kg^−1^), and the lowest one was close to emitter (300 μg kg^−1^). The percentage of 5 + 6-ring compounds increased with the distance from the smelter (14, 16, and 23%); the same regularity was observed for 4-ring PAHs (21, 30, 34%). Slight differences were observed among CP rates at the sites (range 21–40), with the minimum again in B. In grass collected in 2007, Phen/Ant ratio ranged from 3.8 (site A) to 6.0 (site C). After shutting down the smelter, a significant increase in this ratio was observed and it ranged from 9.1 to 27.5 in site C and A, respectively (Table 3).

**Table 3:** Comparison of PAH ratios in grass

Ratio	Year	Site A	Site B				Site C
			Mean	SD	Min	Max	
Phen/Ant	2007	3.8	5.6	1.62	3.9	8.4	6.0
	2010	27.5	20.6	8.01	8.5	31.8	9.1
Flt/Pyr	2007	1.4	1.8	0.34	1.5	2.4	1.5
	2010	1.2	1.5	0.19	1.3	1.9	1.3
BbF/BkF	2007	2.9	2.9	0.35	2.5	3.4	4.0
	2010	1.9	2.0	0.98	0.0	2.1	4.3

The ratio of 4-ring compounds (Flt/Pyr) is often used to characterize PAH sources because it is more conservative than Phen/Ant, which is particularly sensitive to photodegradation of the second (Tobiszewski and Namieśnik 2012). These ratios were slightly differentiated among sites and sampling times and ranged from 1.2 to 1.8.

In grass collected in 2007, the 5-ring compound, BbF/BkF ratio, amounted to 2.9 and 4.0 in sites A and B and site C, respectively. After stopping the smelter operation, the BbF/BkF ratios in site A and B were similar (2.0 approximately) and in site C amounted to 4.3.

### PAHs in spruce needles

It was observed that the PAHs content gradually increased with the age of the needles. However, in the 3-year-old needles, the total amount of PAHs was slightly lower than in the previous year class. In the needles not affected by the smelter emission, the 2- and 3-ring compounds had the highest percentage in the pool of 14 PAHs besides, in the old needles, in which the relative contents of 4- to 6-ring PAH were higher when compared to young needles (Table 4). The CP index for 2- and 3-year-old needles was twice that for in the younger ones.

**Table 4:** PAH contents in spruce needles (μg kg^−1^)—site B (mean ± SD)

PAHs	Current year	1 year old	2 years old	3 years old
Naph	194 ± 28	254 ± 112	238±81	277 ± 151
Fluo	52 ± 22	66 ± 21	58 ± 44	28 ± 9
Phen	78 ± 34	189 ± 26	219 ± 69	174 ± 73
Ant	2.6 ± 0.9	3.4 ± 0.9	5.2 ± 1.5	5.4 ± 2.2
Flt	131 ± 65	235 ± 95	329 ± 99	283 ± 94
Pyr	88 ± 45	185 ± 67	260 ± 81	215 ± 95
BaA	26 ± 13	42 ± 27	77 ± 40	70 ± 36
Chry	76 ± 32	131 ± 77	215 ± 99	195 ± 88
BbF	52 ± 29	93 ± 62	175 ± 96	159 ± 73
BkF	23 ± 13	35 ± 32	72 ± 44	62 ± 28
BaP	36 ± 22	59 ± 42	126 ± 82	108 ± 58
DahA	2.3 ± 1.5	3.8 ± 3.0	11 ± 9.3	7.2 ± 2.9
BghiP	38 ± 15	42 ± 18	91 ± 53	80 ± 35
IcdP	52 ± 24	86 ± 59	159 ± 103	146 ± 69
∑_2-ring PAHs_	194 ± 28 (23%)	333 ± 112 (18%)	296 ± 81 (12%)	384 ± 151 (15%)
∑_3-ring PAHs_	132 ± 57 (15%)	253 ± 6 (18%)	264 ± 26 (14%)	160 ± 66 (11%)
∑_4-ring PAHs_	322 ± 155 (38%)	405 ± 266 (42%)	654 ± 319 (43%)	542 ± 312 (42%)
∑_5-ring PAHs_	113 ± 66 (13%)	93 ± 139 (13%)	221 ± 231 (19%)	223 ± 161 (19%)
∑_6-ring PAHs_	90 ± 40 (11%)	74 ± 78 (9%)	140 ± 156 (12%)	154 ± 104 (13%)
∑ _PAHs_	850	1424	2035	1810
CP	93 ± 47	133 ± 81	276 ± 169	241 ± 117

(%) Percentage values (*m*/*m*)

**Fig. 2:**
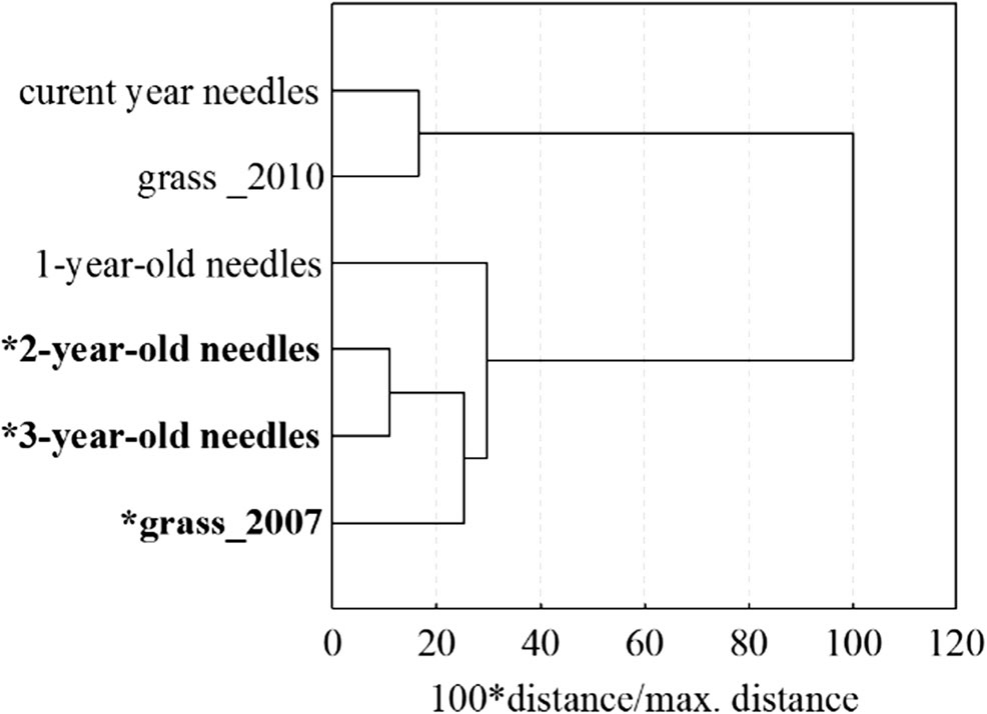
Results of agglomeration of the examined objects. The 2- and 3-ring PAHs were excluded from the analysis (*grew when the aluminum smelter was operating)

The comparison of PAHs concentration in site B in the current year spruce needles and in grass after shutting down the smelter showed that the amount of PAHs in spruce needles was twice as high as in the grass (Tables 2 and 4). A dendrogram was used to examine similarity between plant samples affected and non-affected by emissions, from the smelter (in site B). Great similarity was found between 2- and 3-year-old needles, which were grown when the aluminum smelter was operating, and between non-affected current year, needles and grass were collected in 2010 (Fig. 2). A smaller similarity was found between 1-year-old needles and 2- and 3-year-old needles. It may be caused by the same PAH deposition on the aforementioned needle age classes, which took place after the steelworks shut. It also cannot be ruled out that some of the contaminants on the 2- and 3-year-old needles were not adsorbed by epicuticular waxes and redeposited on the 1-year-old needles.

### PAHs in soil

The total PAHs found in 2007 in soil close to emitter was equal to 20,955 μg kg^−1^ (Table 5). Two- to 4-ring (high/mean volatility) and 5 + 6-ring (low volatility) PAHs had equal shares in the pool; Flt and Pyr from the first group, and BbF and IcdP for the second, had the maximum percentages (> 10% in the PAH distribution). The CPI index rate reached 4867.

**Table 5:** Contents of PAHs in soil (μg kg^−1^)

Year	Site A			Site B				Site C
				Mean^c^	SD	Min	Max	
2007^a^	Naph	105		11	19	(0.9)	45	66
	Fluo	31		2.5	2.4	(0.9)	6.0	9.1
	Phen	1669		119**	50	74	190	81
	Ant	226		6.3	4.7	2.0	14	4.4
	Flt	3008		137**	58	73	216	131
	Pyr	2384		103**	45	58	171	80
	BaA	1634		65**	24	44	99	46
	Chry	1565		107**	52	64	191	80
	BbF	2408		120*	84	38	227	70
	BkF	1194		54*	35	19	96	28
	BaP	2082		83*	56	31	149	65
	DahA	544		22**	7.5	15	34	11
	BghiP	1853		102**	46	57	169	48
	IcdP	2252		153*	99	69	309	55
	∑_2-ring PAHs_	105	(1%)	11 (2%)	19	(0.9)	45	66 (9%)
	∑_3-ring PAHs_	1926	(8%)	128** (12%)	55	78	207	94 (12%)
	∑_4-ring PAHs_	8591	(41%)	413** (39%)	173	238	658	337 (44%)
	∑_5-ring PAHs_	6228	(30%)	278** (24%)	178	103	470	174 (22%)
	∑_6-ring PAHs_	4105	(20%)	255** (23%)	145	126	478	103 (13%)
	∑ _PAHs_	20,955		1085**				774
	*CP*	4867		235**	126	110	393	139
2010^b^	Naph	94		2.9	4.0	(0.9)	8.3	17
	Fluo	104		(0.9)	(0.9)	(0.9)	(0.9)	(0.9)
	Phen	717		31**	16	19	58	41
	Ant	160		(0.9)	(0.9)	(0.9)	(0.9)	(0.9)
	Flt	1266		35**	15	22	59	44
	Pyr	944		27**	11	16	45	31
	BaA	610		17**	9.4	8.8	33	18
	Chry	546		18**	8.2	12	32	24
	BbF	731		30*	21	15	66	29
	BkF	370		11*	6.9	5.7	23	11
	BaP	713		21*	14	11	45	22
	DahA	102		2.3**	4.3	(0.9)	10	3
	BghiP	604		22**	16	11	49	21
	IcdP	914		32*	23	15	71	31
	∑_2-ring PAHs_	94 (1%)		2.9 (2%)	4.0	(0.9)	8.3	17 (6%)
	∑_3-ring PAHs_	981 (13%)		31** (13%)	16	19	58	41 (14%)
	∑_4-ring PAHs_	3366 (43%)		98** (40%)	44	59	169	117 (40%)
	∑_5-ring PAHs_	1916 (24%)		65** (24%)	45	33	144	65 (22%)
	∑_6-ring PAHs_	1518 (19%)		54** (21%)	38	26	121	52 (18%)
	∑ _PAHs_	7875		251**				292
	CP	1640		55**	37	27	118	54

^a^Samples affected by emission from aluminum smelter

^b^Samples collected after emission period

^c^Statistical significance between different years (*n* = 7, *t* test *p < 0.05, **p < 0.01, ***p < 0.001)

(%) Percentage values (*m*/*m*)

Decrease of the total PAHs in soil along with the distance was observed. The mean concentration of PAHs in the area of 0.8 km from the smelter accounted for 1085 μg kg^−1^, while in the site C, it reached 774 μg kg^−1^. Changes also occurred in the PAH profiles. In the area closer to the smelter (site B) and in site C 5- to 6-ring compounds, it contributed for 47 and 35% of the total PAHs, respectively. In both sites, the same compounds as in the soil collected close to the emitter prevailed in the sum of PAHs. The CP index rate was equal to 235 and 139, respectively.

After closing the smelter, the PAH concentration in soil amounted to 7875, 251, and 292 μg kg^−1^ in sites A, B, and C, respectively. It was reflected in the changes of the CP index, the values of which amounted to 1640, 55, and 54, respectively. The slight decrease in the share of 5- and 6-ring classes in the total PAHs near the emitter and the increase in this share in site C were observed. In all sites, the maximum concentrations were reached by Flt for the 2–4-ring class and by IcdP for the 5–6-ring class (Table 5).

## Discussion

Newly grown out grass may be regarded as a bioindicator of PAH emission in a given year. So far, grass has been hardly used as a bioindicator for PAHs due to its smooth surface and a low level of lipids. Concentrations of PAHs in grass near the oil refinery in Zelzate, Belgium (Bakker et al. 2000), in grass affected by the fire of propylene factory in England, and in grass in urban area (Meharg et al. 1998) were 1900, 2400, and 153 μg kg^−1^, respectively. All these values are significantly lower than the ones found near the aluminum smelter (site A) (Table 2). In grass from all sites, the highest concentrations were observed for Fluor, Chr, and BbF. The same pattern was found in the air sampled near the aluminum smelter (using the Söderberg method) in Quebec, Canada (Roussel et al. 1992). After the smelter shutdown in all sites Naph, Phen, Fluo, and Pyr were the compounds with the highest concentration in the grass observed. The same set of concentrations was found in the urban air in Sarajevo and Tuzla, Bosnia, and Herzegovina (Škarek et al. 2007). When the investigated area was no longer affected by the emissions from the smelter, the CP index decreased 2–3 times in sites B and C, and more than 40 times in the site A. Despite that, these values were significantly higher than those found in leaves of birch growing in remote sites of Poland (peat bogs) (Mętrak et al. 2016) and calculated for needles of spruce growing in the Silesian Voivodeship landscape parks and other Polish national parks (Borgulat et al. 2018) with mean CP values reaching 7.8 and 6.3, respectively.

PAH ratios have been widely used as a tool for identifying the emission sources and assessing the role with regard to pollution. In low-temperature processes (e.g., wood burning), low molecular weight PAHs are usually formed while in high-temperature processes, such as fuel combustion in engines, higher molecular weight PAH compounds are emitted (Mostert et al. 2010; Tobiszewski and Namieśnik 2012). The problem with applying molecular ratios in source identification is the chemical and biological alterations of PAHs (Galarneau 2008). Lighter PAHs usually occur in the gaseous phase and can be easily transported on long distances, whereas heavier PAHs are most often bound on particles and their range is shorter.

The ratio of light 3-ring compounds—Phen/Ant—is sensitive to environmental changes and its values for the identification of particular processes lie within a narrow range, which makes it hard to use (Tobiszewski and Namieśnik 2012); thus, Phen/Ant is more useful for petrogenic-pyrogenic discriminations (Stogiannidis and Laane 2015). According to Budzinski et al. 1997 and Stotigandis & Laane 2015, the ratios of Phen/Ant < 15 indicate the dominance of pyrolytic sources (< 5 according to Neff et al. 2005), such as fuel combustion or other high-temperature processes and such values were found for grass in the period of the smelter operation. After its shutdown, the change in the ratio was observed in all sites showing the effect of mixed sources (values 30 > Phen/Ant > 10) (Stogiannidis and Laane 2015).

Coal combustion usually yields values of 4-ring compounds—Fla./Pyr—greater than 1 (Gschwend and Hites 1981; Sicre et al. 1987). Similarly, Flt/Pyr > 1 occurs for coke oven tars and other pyrogenic materials produced at relatively high temperatures (Costa et al. 2004; Saber et al. 2006; Stogiannidis and Laane 2015). Slight differences in this ratio between sites and time of sampling show that the investigated area has been influenced by the PAH deposition originated from fuel combustion.

The pair of 5-ring BbF/BkF is very stable but follows the general trend, which is an increase from the source to the distant point. The same phenomenon was found in our investigation. Moreover, our results comply with the results of investigation carried out in the vicinity of other aluminum smelters. Air samples collected in the stack immediately after going through the clean-up system in the Söderberg aluminum smelter in Canada showed a mean BbF/BkF value of 3.0, whereas in the urban monitoring station located 2.5 km from the smelter, this ratio was 3.6 (Aubin and Farant 2000). On the basis of the investigation carried out in the Cheasapeake Bay region, Dickhut et al. 2000 proposed similar Bbf/BkF ratio (2.5–2.9) as a distinctive for the operation of the aluminum smelters. The values of the ratio found after stopping the smelter operation suggest the influence of mixed sources of PAHs from coal combustion and automobiles for which the values of 3.5–3.9 and 1.1–1.5, respectively, were proposed (Dickhut et al. 2000).

There are a lot of reports on the increase in accumulation of PAHs with the age of needles (Brorström-Lundén and Löfgren 1998; Piccardo et al. 2005; Mętrak et al. 2016) but they refer mainly to three age needle classes (current year, 1- and 2-year-old) and there is no information on the older needle ones. The observed decrease in the amount of PAHs in 3-year-old needles in relation to 2-year-old ones is likely due to the effect of various external stresses which may cause important reductions or alterations of the wax layers simulating the natural process of needle aging and leading to an impotent decrease of uptake rates. Moreover, degradation and loss of the cuticle leads to the loss of previously accumulated compounds (Piccardo et al. 2005). It may be also due to “saturation” of wax layer by the deposited contaminants (Staszewski et al. 1994).

Distinct PAH profiles were observed for needles growing in the period of the smelter activity and after its closing. The analysis of individual PAH concentration shows the prevalence of heavier PAHs in the older needles. Above 70% share of 5 to 6-ring PAHs in needles subjected to smelter emission was observed. It was not the case of more volatile PAHs, whose profiles suggest the inflow of compounds from the urban area and more distant power stations. The differences in the amount of PAHs (after the smelter shutdown) taken up by needles and grass are caused by their aerodynamic roughness. The different lipid content in the leaves is also likely to be a reason of this difference. Although the lipid content was not determined in this experiment, the literature data indicate that grass has a lipid content of approximately 0.3–0.8% (Bohme et al. 1999). For spruce needles, the total lipid content was estimated as 6.4% (Müller et al. 1994).

Cluster analysis (Fig. 2) of 4–6-ring PAHs accumulated in the plant material collected in the period of the smelter operation and after its closing showed similarity between spruce and grass in given periods as well as differences in PAH profiles between needles subjected to emissions from the smelter and those collected after the smelter closing.

The high level of PAHs in the soil close to the emitter is typical of the areas located in the vicinity of industrial plants like a chemical plant in Shanxi, China—35,400 μg kg^−1^ (Jiao et al. 2017), an oil-shale thermal treatment plant in Estonia—12,390 ± 9810 μg kg^−1^ (Trapido 1999), and blast furnace plant in Hoogovens, Netherlands—45,900 μg kg^−1^ (Van Brummelen et al. 1996). The same regularity in the PAH profile near the emitter was found in forest soils affected by the emission of the aluminum plant near Ziar in Central Slovakia (Wilcke et al. 1996). The decrease in the share of 5 + 6-ring compounds and in the CP index determined in the soil reflects differences in the range of individual compound transport. The heavier particle-bond PAHs are deposited faster than the gaseous ones. Such regularity was found also in other studies carried out in the vicinity of aluminum smelters (Wilcke et al. 1996; Aubin and Farant 2000; Rodriguez et al. 2012).

Two years after closing the smelter, the PAHs concentrations and the CP index declined significantly. Changes in PAHs concentrations in the soil near the emitter after the smelter shutdown allow us to contribute to the general discussion about the rate of PAH degradation in soil. However, it should be kept in mind that after sampling the soil in 2007, it had been influenced by the emissions from the smelter over 2 years and then it was affected along 2 consecutive years by relatively low deposition of PAHs, as a grass analysis in 2010 shows. The rough estimates of PAH half-lives in the organic layer of the soil near the blast furnace plant range from 2 to 4 months for Flt, 5–10 months for Fluor, 4–8 months for Pyr, 7–13 months for BaA, 8–13 months for Chry, 1.9–3.3 years for BbF, 11 to 19 months for BkF, and 1.5–2.7 years for BaP (Van Brummelen et al. 1996). The data drawn from a field experiment where sewage sludge containing different concentrations of PAHs was applied to field soils suggest much longer half-lives, and the average half-lives estimated for four and higher ring PAHs ranged from 8 to 17 years (Wild et al. 1991).

**Fig. 3:**
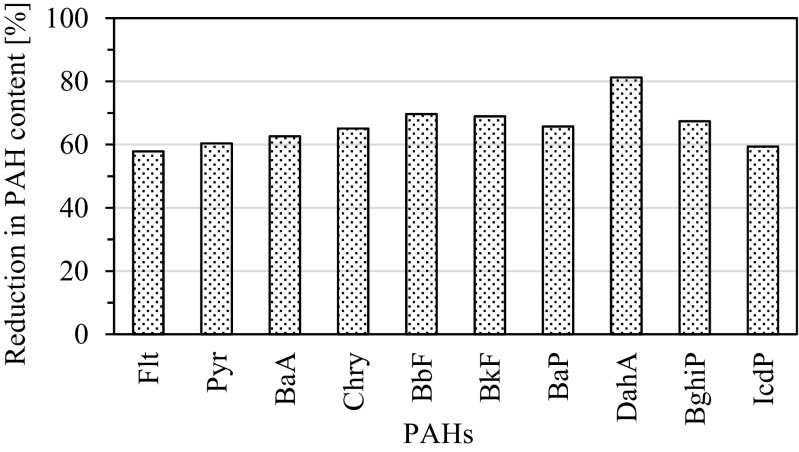
Reduction (*R*) of 4- to 6-ring PAHs content in soil collected close to the aluminum smelter (site A). *R =* (1 − *C*_2010_ × *C*_2007_^−1^) × 100%, where *C*_*x*_ is the content in soil in selected year

The values estimated for the soil close to the aluminum smelter show that concentration of all the analyzed 4- to 6-ring PAHs decreased of 58 to 69% with the exception of DahA where its concentration was reduced by 81% (Fig. 3).

The obtained levels of PAHs in the soil of the sites investigated were compared to the limit values of selected PAHs in soils of agricultural and abandoned lands, i.e., 100 μg kg^−1^ for individual PAHs (Naph, Ant, Phen, Flt, Chry, BaA, BghiP, and BaP) and 30 μg kg^−1^ for BaP (Official Journal of Laws 2002). In both measurement periods, all standardized limit values of PAHs were exceeded in the site near the emitter. The contamination of soil with PAHs decreased with the distance from the smelter and in site B, during the smelter operation, all standardized values were exceeded with the exception of Naph, Ant, and BaA, whereas after closing the smelter, the exceedance of the limit value was not observed. In site C, in the period of the smelter operation, the limit values were exceeded for Flt and BaP. So, according to the definition contained in the Official Journal of Laws (2002), all investigated soils, except for soil in sites B and C after the smelter shutdown, can be considered as contaminated with PAHs.

## Conclusions

In the period of the aluminum smelter operation, the accumulation of PAHs in grass decreased along with the distance from the emitter and the profile of accumulated PAHs was similar to that found in the air near other aluminum smelters. After the smelter shutdown, the profile of PAHs accumulated in grass changed distinctly and was close to the one found in the air sampled in the urban environment. Moreover, the similarity was also stated for 4–6-ring PAH profiles found in grass and spruce needles after closing the smelter. These findings indicate that grass, despite its differences in canopy structure and anatomical and morphological traits compared to trees, responds to changes in the environment burden and hence can be regarded as a good bioindicator of PAHs analogously to previously used leaves of several tree species. The use of four age classes of spruce needles as a biomonitor of PAHs, which showed different PAH profiles in needles growing in the period of the smelter operation and after its closing, demonstrates that it is possible to obtain information regarding the retrospective pollution.

The CP index used by us seems to be a good indicator of environmental hazard posed by PAHs, being a comparative value of hazard if there are no concentration measurements in monitoring stations.

The level of soil contamination shows that the process of aluminum production is an important source of PAHs and the process of self-attenuation in the soil after stopping the production requires years.
